# A Defect Detection Algorithm for Optoelectronic Detectors Utilizing GLV-YOLO

**DOI:** 10.3390/mi16030267

**Published:** 2025-02-26

**Authors:** Xinfang Zhao, Qinghua Lyu, Hui Zeng, Zhuoyi Ling, Zhongsheng Zhai, Hui Lyu, Saffa Riffat, Benyuan Chen, Wanting Wang

**Affiliations:** 1 National “111 Research Center” Microelectronics and Integrated Circuits, School of Science, Hubei University of Technology, Wuhan 430068, China; 2 School of mechanical Engineering, Hubei University of Technology, Wuhan 430068, China; 3 College of Physics and Mechanical Engineering, Hubei University of Education, Wuhan 430205, China; 4 Department of Architecture & Built Environment, The University of Nottingham, Nottingham NG7 2RD, UK

**Keywords:** lightweight model, machine vision, PIN photodiode, surface defect detection

## Abstract

Photodetectors are indispensable in a multitude of applications, with the detection of surface defects serving as a cornerstone for their production and advancement. To meet the demands of real-time and accurate defect detection, this paper introduces an optimization algorithm based on the GLV-YOLO model tailored for photodetector defect detection in manufacturing settings. The algorithm achieves a reduction in the model complexity and parameter count by incorporating the GhostC3_MSF module. Additionally, it enhances feature extraction capabilities with the integration of the LSKNet_3 attention mechanism. Furthermore, it improves generalization performance through the utilization of the WIoU loss function, which minimizes geometric penalties. The experimental results showed that the proposed algorithm achieved 98.9% accuracy, with 2.1 million parameters and a computational cost of 7.0 GFLOPs. Compared to other methods, our approach outperforms them in both performance and efficiency, fulfilling the real-time and precise defect detection needs of photodetectors.

## 1. Introduction

Photodiodes, which function to convert optical radiation into electrical signals, find extensive application across numerous fields [1,2,3]. Among the various types of photodiodes, positive–intrinsic–negative (PIN) photodiodes are particularly esteemed for their exceptional frequency response, high sensitivity, and superior signal-to-noise ratio. These attributes render PIN photodiodes ideal for monitoring and imaging tasks within the visible and infrared wavelength ranges [4,5,6]. Due to these qualities, PIN photodiodes have established themselves as indispensable components in a wide range of fields, including aerospace [7], defense and security, optical communications [8], medical devices [9], and scientific instruments. Despite their simple structure and well-established manufacturing processes, the production of photodiodes is still susceptible to defects caused by factors like manufacturing techniques, environmental conditions, and equipment variability [10,11]. As the semiconductor industry progresses and device dimensions continue to shrink, the demand for higher quality photodiodes has intensified [12]. Consequently, surface defect detection is crucial not only for enhancing the device quality and yield but also for driving innovation in the semiconductor industry.

The need for automated surface defect detection in semiconductor manufacturing has grown significantly. Common defects include foreign particles, fractures, and organic contamination, all of which can compromise device performance [13]. Traditionally, these defects were identified manually by skilled inspectors, a process that is inefficient, error-prone, and costly. Machine vision methods have provided an alternative, offering reliable detection for simple and well-defined defects, but they often fail when addressing complex defect patterns [14,15]. The advent of computer vision technologies has resolved many of these limitations, enabling their application in semiconductor defect detection. For example, Jiabin Jiang et al. developed a U-Net-based convolutional neural network (CNN) with an encoder–decoder architecture to detect surface defects on screen-printed smartphone back glass, achieving over 91% accuracy and a recall rate exceeding 95% [16]. Similarly, Hang Zhang et al. proposed a deep learning-based three-stage approach for TO56 semiconductor laser defect detection, encompassing localization, segmentation, and defect pattern recognition [17]. Shang Wu et al. introduced the Dense Skip Connection U-Net (DSCU-Net), which optimized skip connections between the encoder and decoder to enhance high-order feature integration, effectively addressing defects in semiconductor chip manufacturing [18]. However, these methods are often limited by high computational demands, complex models, and slower detection speeds.

The You Only Look Once (YOLO) framework has gained popularity for its balance of speed and accuracy in object detection [19,20,21]. For instance, Fei Ren et al. introduced the ECA-SimSPPF-SIoU-YOLOv5 algorithm for steel defect detection, achieving a 7.1% improvement in the mean average precision (mAP) compared to the original YOLOv5s model [22]. Moyun Liu et al. developed the LF-YOLO algorithm for detecting welding defects in X-ray images, incorporating an efficient feature extraction (EFE) module that achieved an mAP of 92.9% and a frame-per-second (FPS) rate of 61.5 [21]. Yunchang Zheng et al. proposed the GBCD-YOLO model for high-precision, lightweight, and real-time wood surface defect detection, reporting a 13.45% improvement in the mAP(0.5) and an 11.95% improvement in the mAP(0.5:0.95), along with a 6.25 FPS increase compared to YOLOv5s, while reducing the model parameters by 15.49% [23]. These studies demonstrate the potential of YOLO-based models for surface defect detection in PIN photodiodes.

Despite these advances, limited research has focused specifically on using deep learning for detecting surface defects in PIN photodiodes. In this study, we compiled a labeled dataset tailored to PIN photodiode surface defects and applied diverse data preprocessing techniques to improve the model robustness in practical scenarios. To address the challenges of maintaining detection accuracy while minimizing the model complexity and computational cost, this paper introduces a GLV-YOLO-based optimization algorithm tailored for PIN photodiode surface defect detection. The proposed model outperforms YOLOv8 in terms of accuracy, with fewer parameters and reduced computational requirements.

## 2. Related Work

**Figure 1:**
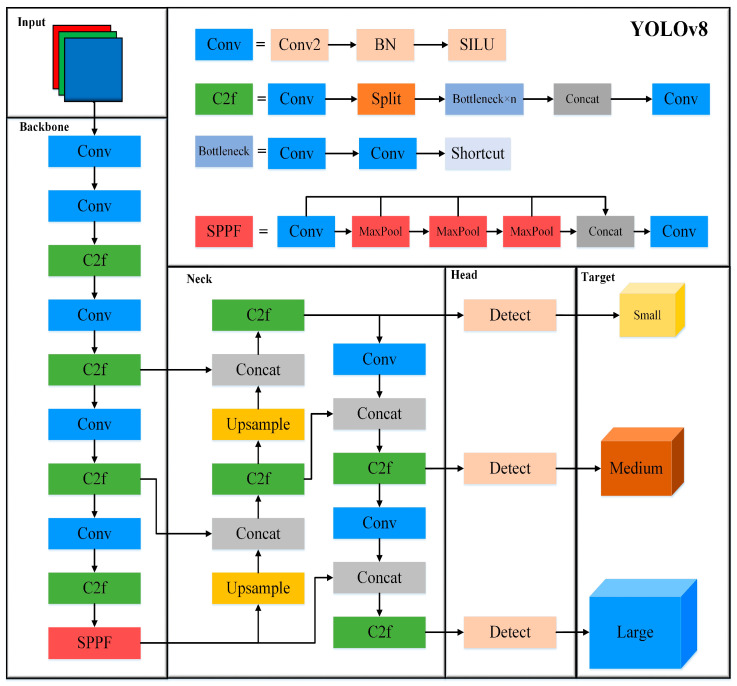
Schematic diagram of YOLOv8 architecture.

One-stage detection models are renowned for their speed and accuracy, with the YOLO series being a prime example of this category. YOLOv8, developed by Ultralytics, represents one of the latest advancements in the YOLO series of object detection algorithms. The YOLOv8 framework is available in five variants, YOLOv8n, YOLOv8s, YOLOv8m, YOLOv8l, and YOLOv8x, ordered by increasing model weight, width, and depth. As illustrated in Figure 1, the overall network architecture of YOLOv8 comprises four main components: the input layer, Backbone layer, Neck layer, and Head layer.

Backbone: YOLOv8 utilizes CSPDarkNet as its Backbone network. The Conv module consists of convolutional layers, Batch Normalization (BN) layers [24], and an SiLU activation function [25]. The convolutional layers extract feature information from the input image, which is then processed through the BN layers to accelerate the network’s convergence. Finally, the SiLU activation function helps the network better adapt to complex data and model more intricate functions. The BN layer operations are detailed in Formulas (1)–(4), which include calculating the sample mean, computing the sample variance, standardizing the data, and applying translation and scaling, where *γ* and *β* are learnable parameters.

(1) $$\mu_{\beta }=\frac{1}{m}\sum_{i=1}^{m}x_{i}$$

(2) $$\sigma_{\beta }^{2}=\frac{1}{m}\sum_{i=1}^{m}{\left(x_{i}-u_{\beta }\right)}^{2}$$

(3) $$x_{i}^{\prime }=\frac{x_{i}-u_{\beta }}{\sqrt{\sigma_{\beta }^{2}+\varepsilon }}$$

(4) $$y_{i}=\gamma x_{i}^{\prime }+\beta =BN_{\gamma ,\beta }(x_{i})$$

The SiLU activation function has a derivative that approaches 1 as the input approaches 0, which allows it to retain more feature information during forward propagation. The computation process for SiLU is shown in Formula (5).

(5) $$SiLU(x)=x*sigmoid(x)=\frac{x}{1+e^{-x}}$$

The C2f module is introduced as a residual connection module to better preserve the feature information of the original image. This module splits the output into two parts. One part is passed directly to the output, while the other part is processed through multiple Bottleneck modules. Finally, the results from both parts are concatenated along the channel dimension and passed through a second convolutional layer (Conv) to obtain the final output. The SPPF module is then used to capture features at different scales.

Neck: This layer adopts the FPN+PAN feature pyramid structure [26], which performs feature fusion across different scale levels. This effectively extracts feature information at various scales, constructing a feature pyramid with rich information across all scales.

Head: The Head consists of three different scale layers that output information such as the object confidence, class scores, and anchor box coordinates for objects of various sizes. Non-Maximum Suppression [27] (NMS) is then applied to obtain the final object coordinates.

**Figure 2:**
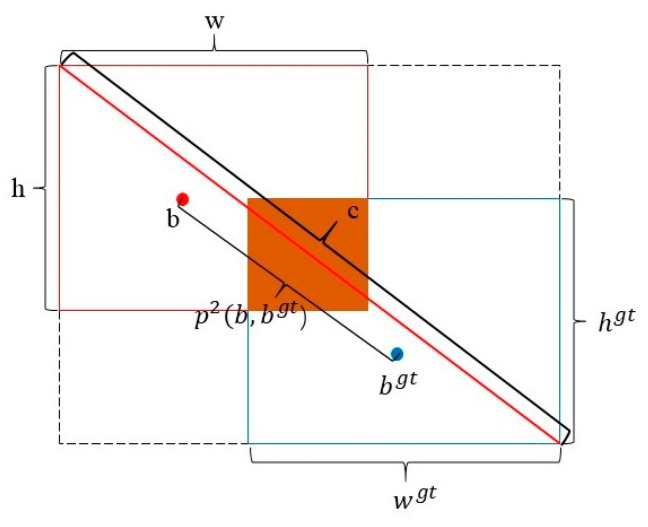
Schematic diagram of the CIoU loss function.

Loss Function: YOLOv8 uses CIoU [28] as the loss function for anchor boxes, as shown in Formulas (6)–(8), where α represents the weight function and ν measures the aspect ratio. A diagram of the CIoU loss is illustrated in Figure 2.

(6) $$\alpha =\frac{v}{(1-IoU)+v}$$

(7) $$v=\frac{4}{\pi^{2}}{\left(\arctan\frac{w^{gt}}{h^{gt}}-\arctan\frac{w}{h}\right)}^{2}$$

(8) $$CIoULoss=1-IoU+\frac{\rho^{2}(b,b^{gt})}{c^{2}}+\alpha v$$

## 3. Proposed Method

**Figure 3:**
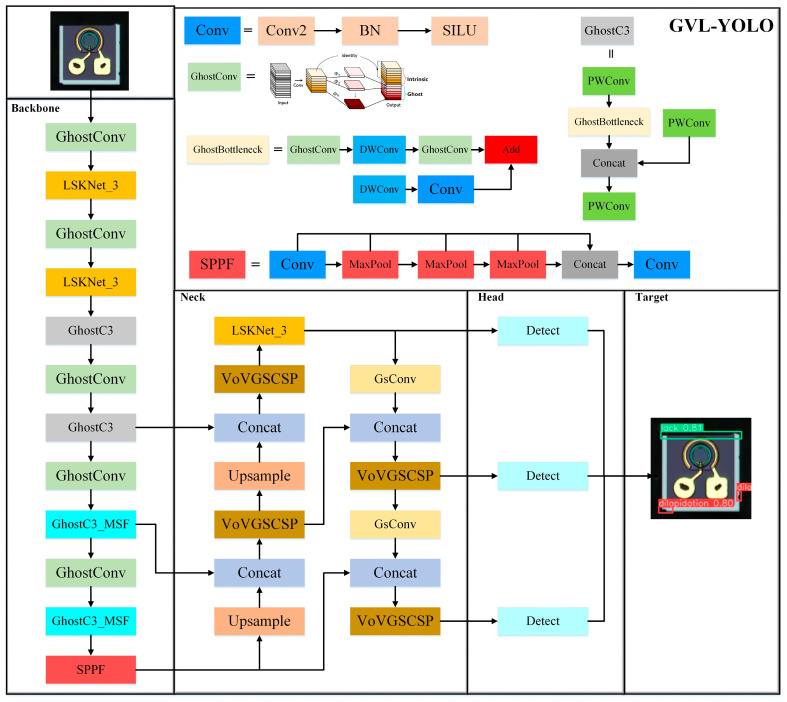
The overall framework of the GVL-YOLO model.

The GVL-YOLO model framework, shown in Figure 3, consists of four main components: the input layer, Backbone layer, Neck layer, and Head layer. The input layer performs image preprocessing before the images are passed into the model. Various augmentation techniques, including geometric transformations, exposure adjustments, field-of-view darkening, and noise addition, are applied to enhance the dataset and improve the model’s robustness.

In the Backbone layer, the GhostConv [29] and GhostC3 models are used to reduce the model’s parameter count and computational complexity, achieving a more lightweight design. For the last two layers, which have more channels, GhostC3_MSF redistributes the weights across these channels to improve feature extraction. Additionally, the LSKNet [30] module is incorporated to capture contextual information at multiple scales, further enhancing the model’s feature extraction capabilities.

The Neck layer uses an FPN+PAN feature pyramid structure [26] to fuse features across multiple scales. This structure efficiently extracts information at various scales and constructs a feature-rich pyramid. Lightweight modules, such as GSConv [31] and VoVGSCSP, replace the original Neck modules, reducing the number of parameters while maintaining accuracy.

Finally, in the Head layer, predictions are made for class labels and anchor box locations. The WIoU loss [32] function is used for the localization loss, which more accurately estimates the model performance and leads to improved predictions with lower loss.

### 3.1. Lightweight Backbone Network

In convolutional neural networks (CNNs), frequent convolution operations often lead to increased model parameters and computational costs. To address this issue, the present study replaced the original YOLOv8 Backbone with the GhostConv and GhostC3 modules, resulting in a lightweight and efficient architecture. The model uses multiple 1 × 1 convolutional kernels instead of the larger kernels from the original network and incorporates depthwise convolutions. This design reduces the number of parameters while improving computational efficiency.

Although the use of depthwise separable convolutions improves the efficiency of the model, it may lead to a loss in accuracy. To strike a balance between model efficiency and accuracy, we adopted a multi-scale selective fusion approach, which redistributes weight information for comprehensive fusion. This approach reduces the model parameters while enhancing accuracy. In the latter half of the Backbone, we designed the GhostC3_MSF (multi-scale selective fusion) network. In this module, we replaced the original GhostBottleneck structure in the GhostC3 module with our proposed GhostBottleneck_MSF structure.

For the input feature layer *F_in_* of the GhostBottleneck_MSF, we first replaced the residual structure in the original YOLOv8 with the GhostBottleneck [29], aiming to reduce the computational complexity using lightweight convolution operations while ensuring no significant performance degradation, thereby preserving the model’s ability to extract spatial features effectively. The output feature maps *FM*_1_ and *FM*_2_ from the GhostBottleneck are then processed through global average pooling and 1 × 1 convolution operations, transforming spatial features into channel-wise representations. These representations are used to generate the channel attention weights *W*_1_ and *W*_2_.

**Figure 4:**
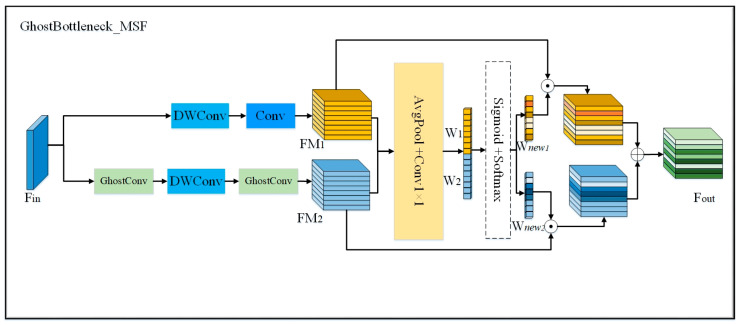
Schematic of the GhostBottleneck_MSF structure.

Next, the weights *W*_1_ and *W*_2_ are concatenated, and the Softmax and sigmoid activation functions are applied to compute the normalized probabilities of the two weights, resulting in the redistributed weights *W_new_*_1_ and *W_new_*_2_. This process enhances the model’s focus on the most important channels, emphasizing relevant features while suppressing less significant ones. The updated weight information is then applied to the feature maps through weighted fusion, yielding refined spatial feature information. Finally, a residual connection is established between the two feature layers to enable the learning of deeper, more abstract features. The specific architecture of the GhostBottleneck_MSF structure is illustrated in Figure 4.

In summary, by replacing the original GhostBottleneck structure in the GhostC3 network with our newly designed GhostBottleneck_MSF, we created the GhostC3_MSF network, which combines the benefits of multi-scale fusion with efficient computation and enhanced accuracy.

### 3.2. Lightweight Modules in the Neck Layer

Within the Neck layer, the feature scales are reduced, concentrating feature information within the channels with minimal redundancy. This allows the model to maintain performance while reducing the number of parameters. In this study, the GSConv and VoVGSCSP lightweight modules were introduced to replace the convolutional components in the Neck layer of the original model. Compared to other lightweight modules, GSConv effectively preserves hidden connections between channels, which reduces the network’s complexity while maintaining high accuracy. This approach balances model performance and speed.

**Figure 5:**
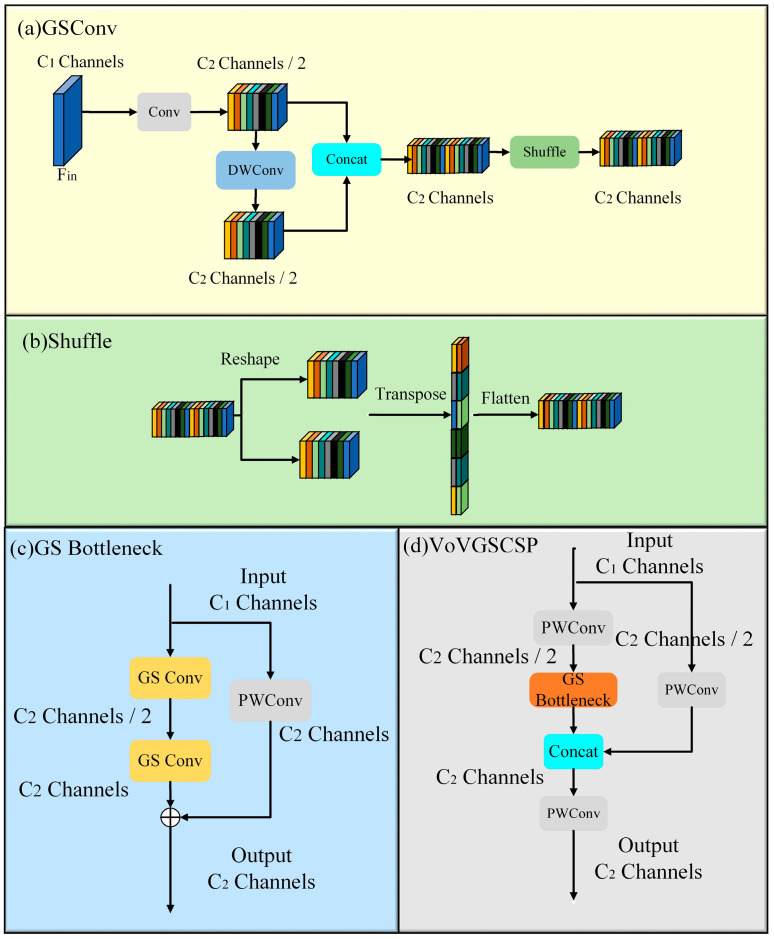
Structure of the GSConv and VoVGSCSP modules.

In the GSConv module, the input has *C*_1_ channels, and the output has *C*_2_ channels. First, a standard convolution operation is applied to the input feature layer *F_in_*, producing a hidden feature layer with *C*_2_/2 channels, which reduces the number of parameters. Next, depthwise separable convolution (DWConv) is applied to the hidden feature layer, keeping the number of channels at *C*_2_/2. Then, a concatenation operation (Concat) is used to merge the results of the standard convolution and depthwise convolution, ensuring the final output has *C*_2_ channels. Finally, a channel shuffle operation is applied to enhance the fusion of information across different channels, improving the model’s ability to extract semantic information. The specific steps are illustrated in Figure 5a,b.

Next, the GSConv modules are used to construct the GSBottleNeck module, as shown in Figure 5c. The module consists of two paths: the first path uses two GSConv layers, with the first outputting half of the target output channels; the second path is composed of a 1 × 1 point-wise convolution (PWConv). The outputs of both paths are then fused through a residual connection to generate an output feature map with the desired *C*_2_ channels.

The final VoVGSCSP module is constructed by combining the GSBottleNeck module with several PWConv layers. In the VoVGSCSP module, the PWConv layers reduce the input channels by half, and the results of the GSBottleNeck module are concatenated with the output from the PWConv layers. This process produces the final feature map with *C*_2_ output channels. The detailed operation is illustrated in Figure 5d.

### 3.3. The Introduction of the LSKNet_3 Attention Mechanism

In the dataset used in this study, many defect types occurred at the edges of the images, making edge detection particularly crucial. To improve the network’s sensitivity to edge defects, larger convolutional kernels were employed to expand the receptive field. A larger receptive field typically enhances the network’s ability to capture global features, enabling it to learn more comprehensive, broader, and semantically richer representations. However, large convolutional kernels pose challenges, including the potential loss of fine details and an increase in the number of parameters.

To overcome these challenges, this study utilized the LSKNet model, which decomposes large convolutional kernels into dilated convolutions with varying sizes and dilation rates. This approach allows the network to generate feature representations with different receptive fields, effectively capturing edge and other defect information. Moreover, the decomposition of large kernels into smaller ones reduces the parameter count, improving the network’s efficiency while maintaining strong semantic information extraction.

The LSKNet_3 network is composed of three key components: the decomposition of large convolutional kernels, spatial feature interaction, and feature-weighted summation. The decomposed convolutional layers are first applied to the input feature map X∈R^C×H×W^, enabling the extraction of contextual information at various scales.

(9) $$U_{0}=XU_{i+1}=F_{i}^{dK}(U_{i})$$

The ith depthwise separable convolution layer, with a kernel size of *K_i_* and a dilation rate of *d_i_*, is denoted by ${\;F}_{i}^{dK}\left(\cdot \right)$.The output of the i + 1th feature layer is represented by *U_i_*_+1_. A subsequent 1 × 1 convolution with the dilation rate $F_{i}^{1\times 1}\left(\;\right)$ is applied to divide the input convolution channels into *N* groups, facilitating the extraction of distinct features from different receptive fields.

(10) $$U_{i}=F_{i}^{1\times 1}(U_{i})$$

$W_{i}^{1\times H\times W}$ represents the features at the ith scale.

To direct the network’s focus toward the most relevant spatial regions, features at multiple scales are extracted using three distinct receptive fields and concatenated along the channel dimension. Then, global average pooling and global max pooling operations are performed along the channel axis to compress the global channel information.

(11) $$U=\mathrm{Concat}(U_{1}\ldots U_{i})$$

(12) $${\mathrm{SA}}_{\mathrm{avg}}\in R^{1\times H\times W}=\mathrm{AvgPool}(U)$$

(13) $${\mathrm{SA}}_{\max}\in R^{1\times H\times W}=\mathrm{MaxPool}(U)$$

To facilitate the interaction between the two types of spatial information, their respective feature layers are concatenated. A convolutional layer, followed by a sigmoid function, is then used to compute the corresponding weight $W_{i}^{1\times H\times W}$ for each scale. These N weights are applied to the N feature layers, where they are used for weighted feature fusion.

(14) $$W_{i}^{1\times H\times W}=\mathrm{sigmoid}({F_{i}}^{2\to N}{(\mathrm{Concat}(\mathrm{SA}}_{\mathrm{avg}}{,\mathrm{SA}}_{\max})))$$

(15) $$S\in R^{C\times H\times W}=F(\sum_{i}^{N}W_{i}^{1\times H\times W}\cdot U_{i})$$

The final output is obtained as the point-wise, element-wise product between the input feature $X\in R^{C\times H\times W}$ and the output weight layer $Y\in R^{C\times H\times W}$.

**Figure 6:**
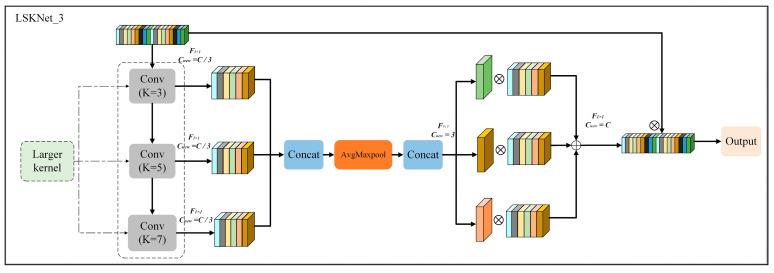
Structure of the LSKNet_3 model.

The LSKNet_3 model is illustrated in Figure 6.

### 3.4. Loss Function

The CIoU loss function considers the overlap between the predicted and ground truth bounding boxes, the distance between their centers, and the aspect ratio. However, low-quality samples in training datasets may be penalized more heavily due to geometric factors such as the distance and aspect ratio, which can negatively impact the model’s generalization ability. To address this issue, the WIoU loss function introduces a distance-aware mechanism (RWIoU) to reduce the influence of these geometric factors. Furthermore, while the CIoU loss function increases the computational complexity, the WIoU loss function effectively reduces the computational burden, thereby enhancing the detection speed. The WIoU loss function is described in Equations (16) and (17).

(16) $$L_{WIoU}=R_{WIoU}L_{IoU}$$

(17) $$R_{WIoU}=\exp\left(\frac{{\left(x-x_{gt}\right)}^{2}+{\left(y-y_{gt}\right)}^{2}}{\left({W_{g}}^{2}+{H_{g}}^{2}\right)}\right)$$

## 4. Experiments and Analysis

### 4.1. Dataset

The primary detectors used in optical communication are PIN photodetectors and avalanche photodiodes (APDs). This study collected a dataset comprising nine different defect types occurring during the production of PIN photodiodes. The types and corresponding sample counts of photodiode surface defects collected in this study are presented in Table 1. The images in the dataset had a resolution of 237 × 239 pixels. In total, 3450 images of surface defects on PIN photodiodes were collected and split into training, validation, and test sets in an 8:1:1 ratio. Specifically, the dataset contained 2760 images for training, 345 for validation, and 345 for testing. The dataset was collected under a microscope, and the training set was labeled using a manual annotation method.

**Figure 7:**
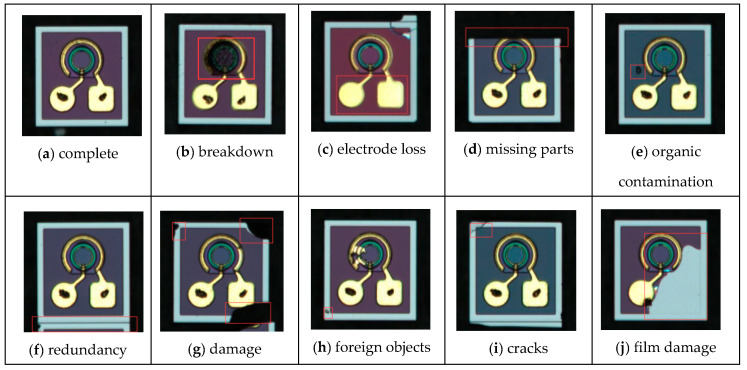
Examples of various diode defect types.

The defects in the diodes were categorized as follows: an image of a complete photodetector is shown in Figure 7a, while other defect types included breakdown (Figure 7b), electrode loss (Figure 7c), missing parts (Figure 7d), organic contamination (Figure 7e), redundancy (Figure 7f), damage (Figure 7g), foreign objects (Figure 7h), cracks (Figure 7i), and film damage (Figure 7j). A sample of the collected images depicting these defects is shown in Figure 7.

### 4.2. Image Preprocessing

**Figure 8:**
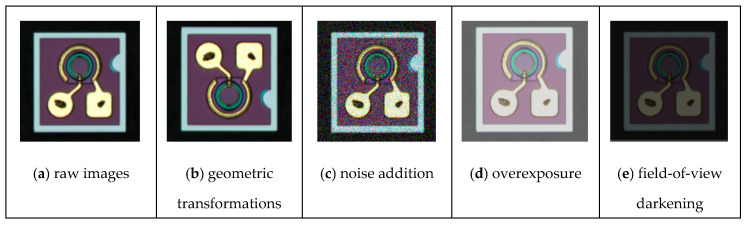
Examples of Image Enhancement Techniques.

The dataset was collected under normal conditions with the microscope sampling equipment functioning properly, resulting in high-quality samples. However, to simulate potential real-world issues, such as noise and variations in the field of view, we applied several image enhancement techniques to artificially expand the dataset. These techniques included (b) geometric transformations, (c) noise addition, (d) field-of-view brightening, and (e) field-of-view darkening. These preprocessing steps aimed to improve the robustness of the model, enabling it to handle the complex scenarios and variations often encountered during real-world defect detection. Figure 8 illustrates examples of these preprocessing steps.

### 4.3. Computational Environment and Simulation Parameters

The computational environment used for model training and evaluation consisted of the following hardware and software components: a Windows 10 operating system, Intel(R) Xeon(R) W-2235 CPU @ 3.80 GHz, NVIDIA RTX A2000 GPU (12GB), 64 GB of RAM, PyTorch 2.2.2 with CUDA 12.1, and Python 3.10. The simulation parameters are detailed in Table 2.

### 4.4. Model Evaluation Metrics

To objectively evaluate the performance of defect detection tasks across various defect types, the following metrics were considered: P (precision), R (recall), the mean average precision at an IoU of 0.5 (mAP@0.5), the mean average precision at an IoU of 0.5–0.95 (mAP@0.5–0.95), and the number of model parameters. The precision represents the proportion of true positive samples among all detected targets; the recall measures the proportion of true positives correctly identified from all actual positive samples; the mAP@0.5 refers to the average precision (AP) at an Intersection over Union (IoU) threshold of 0.5, where the IoU is the ratio of the overlap between anchor boxes; and the parameters refer to the number of parameters in the model. The specific formulas are provided in Equations (18)–(21).

(18) $$P=\frac{TP}{TP+FP}$$

(19) $$R=\frac{TP}{TP+FN}$$

(20) $$AP=\int_{0}^{1}P(R)dR$$

(21) $$mAP=\frac{\sum_{i=1}^{N}AP_{i}}{N}$$

In this context, TP denotes true positives, where the label is positive and the prediction is also positive; FP represents false positives, where the label is negative but the prediction is positive; and FN refers to false negatives, where the label is positive but the prediction is negative. AP indicates the average precision for each class, N is the number of classes, and A$P_{i}$ represents the average precision across all classes.

### 4.5. The Experimental Results of the Model Architecture

To validate the effectiveness of the proposed improvements, experiments were conducted using different model architectures on the baseline model. First, GhostNet was used to replace the Backbone network for feature extraction. Next, the GSConv and VoVGSCSP layers were introduced in the Neck module. Finally, the LSKNet attention mechanism was applied. The results of the ablation study are presented in Table 3. The original YOLOv8 model achieved an mAP of 95.8%. After introducing GhostNet into the Backbone (Model 1), the model parameters decreased by 22.5%, with a slight reduction in the mAP. In Model 2, the addition of GSConv and VoVGSCSP layers in the Neck module led to a 13.3% reduction in the model parameters. The introduction of the LSKNet attention mechanism and changes in the network structure resulted in a slight increase in the model parameters, but the mAP improved by 3.2%. However, this led to an increase in the computational complexity. The improved YOLOv8 model demonstrated an accuracy increase of 0.8%, a recall rate improvement of 4.9%, an mAP@0.5 increase of 3.1%, and an mAP@0.5–0.95 increase of 10.5%. These results indicate that the proposed modifications significantly enhanced the performance of the YOLOv8 model in detecting surface defects in optoelectronic detectors.

**Figure 9:**
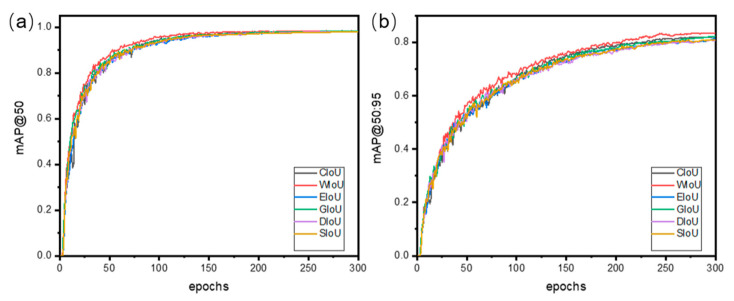
Comparison of Various IoU Loss Functions. (**a**) Curve of mAP@50. (**b**) Curve of mAP@50-90.

To evaluate the advantages of the proposed WIoU loss function over other commonly used loss functions, a comparison was made between the WIoU and SIoU, CIoU, EIoU, GIoU, and DIoU loss functions. As shown in the experimental results in Figure 9a for the mAP@50 and Figure 9b for the mAP@50:95, the WIoU loss function consistently outperformed the others in terms of the average precision after 25 training epochs. Specifically, as indicated in Table 4, the WIoU loss function achieved a 1.5% improvement over CIoU, a 2.6% improvement over SIoU, and a 2% improvement over EIoU at an mAP@50:95.

### 4.6. Results Analysis

**Figure 10:**
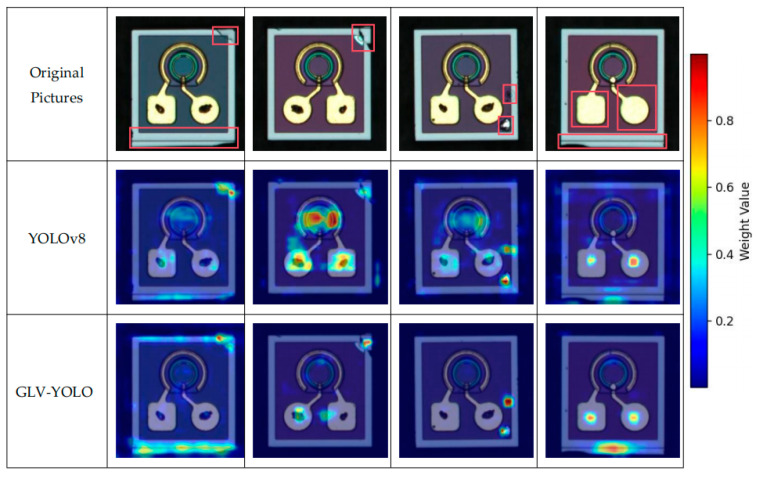
Comparison of heatmaps from different models.

Figure 10 depicts a comparison of heatmaps for different models. Although the YOLOv8 model focuses on surface defect locations, it also assigns weights to irrelevant areas. This results in false positives and missed detections. In contrast, the GLV-YOLO model more effectively targets defect locations, assigns higher weights to these areas, and reduces the weights given to irrelevant regions. Consequently, GLV-YOLO has a lower rate of false positives and missed detections compared to YOLOv8. In these heatmaps, the colors represent the weight assigned by the model to each region, where cold colors (e.g., blue) correspond to areas with lower weights, indicating less importance or attention, and warm colors (e.g., red and yellow) represent areas with higher weights, suggesting a greater significance or focus for defect detection.

**Figure 11:**
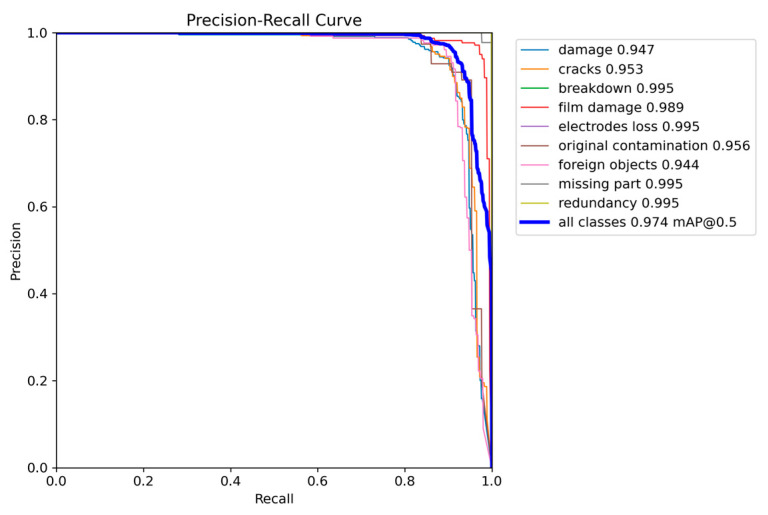
Accuracy–recall results of GLV-YOLO.

**Figure 12:**
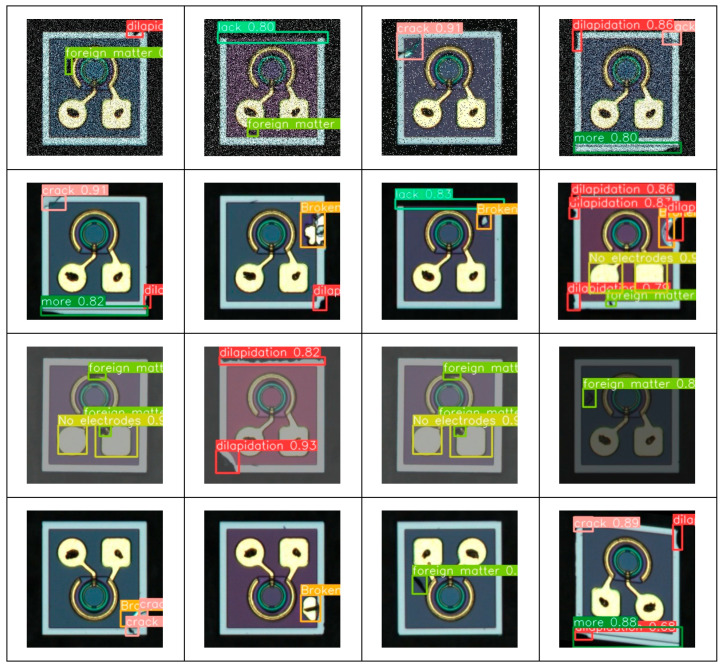
Defect detection results of the proposed model.

The accuracy–recall curves for the nine types of defects detected by the proposed model are shown in Figure 11, with an average accuracy of 98.6%. These results demonstrate that the model exhibits strong defect detection capabilities for optoelectronic diode surfaces. Figure 11 presents the defect detection results of the proposed model under various challenging environments, including noisy, normal, field-of-view brightness variation, and geometric transformation conditions. As shown in Figure 12, the proposed algorithm was able to accurately identify various defect categories on the surface of optoelectronic diodes across different detection environments.

### 4.7. Comparison with Other Models

In this section, we compare the proposed model with several other models, including Yolov5s, Yolov7-tiny, Yolov8n, Yolov8s, and Yolov3tiny. The results clearly show that our proposed model outperforms these methods. On the dataset of optoelectronic detector defects, our algorithm achieved higher accuracy than Yolov5s, Yolov7-tiny, and Yolov8n. Although the mAP value of our model was comparable to that of Yolov8s, our algorithm had fewer parameters and a lower computational cost. In Table 5, the mAP and computational costs for different models are presented. Our proposed algorithm achieved high accuracy with a small computational cost, reaching an average precision of 98.9% with a computational load of 7 GFLOPs. From Table 5, it is evident that our algorithm had 2.18 million parameters and a weight size of 4.7 MB. Overall, the proposed algorithm maintained high precision with fewer parameters, allowing for the efficient and accurate detection of surface defects on optoelectronic diodes. However, the FPS of our model was 51, which is 19 frames lower than that of the original model.

## 5. Conclusions

This study presents a lightweight detection method, GVL-YOLO, designed to efficiently identify small surface defects on optoelectronic diodes. The method integrated the LSKNet_3 attention mechanism into the original model to enhance the extraction of contextual information. By using convolution kernels of varying sizes, it sampled different receptive fields, effectively capturing the context across a range of defect sizes. Furthermore, the inclusion of lightweight GSConv and VoVGSCSP modules reduced the model’s parameters from 3.1 million to 2.18 million, representing a 30.5% reduction compared to the original algorithm. The improved method boosted accuracy by 3.1%, while reducing the computational cost by 20%, ensuring precise defect detection on optoelectronic detectors’ surfaces. When compared to mainstream algorithms, the proposed method demonstrated significant improvements, making it more suitable for real-time monitoring and high-precision detection. Additionally, the model’s weight file size decreased from 5.94 MB to 4.71 MB, further enhancing its compactness. Overall, the GVL-YOLO model represents a substantial advancement in surface defect detection for optoelectronic diodes, with broad applications in real-time monitoring and quality control in semiconductor manufacturing. The findings demonstrate the feasibility of implementing more efficient, lightweight models for industrial use, and future work will focus on further optimizing the model depth and detection speed.

## Figures and Tables

**Table 1:** Defect types and sample counts.

Defect Type	Sample Count
breakdown	60
electrode loss	690
missing parts	360
organic contamination	480
redundancy	585
damage	3384
foreign objects	1782
cracks	1755
film damage	1947

**Table 2:** Simulation parameters.

Simulation Parameters	Parameter Values
Epochs	300
Batch size	32
Learning rate	0.01
Optimizer	SGD

**Table 3:** Experimental results using different model architectures on our dataset.

Model	GhostNet (Backbone)	GsConv + VoV (Neck)	LSKNet_3	mAP0.5 (%)	mAP0.5–0.95 (%)	Param.	P (%)	R (%)	GFLOPs
YOLOv8n	×	×	×	95.8	72.9	3,157,200	95.6	90.9	8.9
Model 1	√	×	×	94.9	70.6	2,445,167	93.2	89.3	7.2
Model 2	×	√	×	96.3	76.3	2,737,107	94.3	92.6	7.9
Model 3	×	×	√	98.5	84.1	3,199,881	96.9	96.6	9.0
Model 4	√	√	×	95.3	72.8	2,169,671	95.4	90.3	6.3
Model 5	√	√	√	98.9	83.4	2,187,390	96.4	97	7.0

**Table 4:** Comparison of the loss functions in the experimental study.

Loss Function	CIoU [28]	WIoU	EIoU [33]	GIoU [34]	DIoU [35]	SIoU [36]
mAP@50 (%)	98.2	98.9	98.2	98.2	97.9	98
mAP@50:95 (%)	81.9	83.4	81.4	82.3	80.7	80.8

**Table 5:** Comparison of different models.

Model	mAP0.5 (%)	mAP0.5–0.95 (%)	FLOPs (G)	Parameters (Million)	Weights_file (Mb)	FPS
Yolov3tiny	91.8	58.6	19.1	12.1	11.7	140
Yolov5s	91.1	59.2	16	7.0	13.6	24
Yolov7tiny	89.2	54.4	3.46	6.03	11.7	55
Yolov8n	95.8	72.9	8.9	3.16	5.94	65
Yolov8s	98.7	83.7	28.7	11.2	21.4	60
GLV-YOLO	98.9	83.4	7.0	2.18	4.7	46

## Data Availability

The data are available on request from the authors.
